# Community-engaged research in translational science: Innovations to improve health in Appalachia

**DOI:** 10.1017/cts.2021.862

**Published:** 2021-10-07

**Authors:** Scott D. Rhodes, Parissa J. Ballard, Keena R. Moore, Karen Klein, Isaiah Randall, Michael Lischke, Aaron T. Vissman, Eugene J. Lengerich, Stephanie S. Daniel, Joseph A. Skelton

**Affiliations:** 1 Department of Social Sciences and Health Policy, Wake Forest School of Medicine, Winston-Salem, NC, USA; 2 CTSI Program in Community-Engaged Research, Wake Forest School of Medicine, Winston-Salem, NC, USA; 3 Department of Family and Community Medicine, Wake Forest School of Medicine, Winston-Salem, NC, USA; 4 Clarus Editorial Services, Santa Fe, NM, USA; 5 The Northwest Area Health Education Center, Winston-Salem, NC, USA; 6 Talbert House, Cincinnati, OH, USA; 7 Penn State College of Medicine, Hershey, PA, USA; 8 Department of Pediatrics, Wake Forest School of Medicine, Winston-Salem, NC, USA

**Keywords:** Health equity, community engagement, translation

## Abstract

Health disparities between Appalachia and the rest of the country are widening. To address this, the Appalachian Translational Research Network (ATRN) organizes an annual ATRN Health Summit. The most recent Summit was held online September 22–23, 2020, and hosted by Wake Forest Clinical and Translational Science Institute in partnership with the Northwest Area Health Education Center. The Summit, titled “Community-Engaged Research in Translational Science: Innovations to Improve Health in Appalachia,” brought together a diverse group of 141 stakeholders from communities, academic institutions, and the National Center for Advancing Translational Science (NCATS) to highlight current research, identify innovative approaches to translational science and community-engaged research, develop cross-regional research partnerships, and establish and disseminate priorities for future Appalachian-focused research. The Summit included three plenary presentations and 39 presentations within 12 concurrent breakout sessions. Here, we describe the Summit planning process and implementation, highlight some of the research presented, and outline nine emergent themes to guide future Appalachian-focused research.

## Introduction

The Appalachian Region covers 205,000 square miles, from New York to Mississippi; other states in Appalachia are Pennsylvania, Maryland, Ohio, West Virginia, Virginia, Kentucky, North Carolina, Tennessee, South Carolina, Georgia, and Alabama. More than 25.6 million people live in the region, and 42% of the region’s population is rural, compared with 20% of the US population [1,2]. The average household income in Appalachia is 80% of the national average [2], and health disparities are widening [3,4]. The region has a higher percentage of census tracts identified as food deserts [5]. Obesity, smoking, substance misuse, and physical inactivity are all more prevalent in Appalachia than in the USA overall [6–8]. Not surprisingly, life expectancy is 2.4 years shorter than in the rest of the USA [4]. Yet, Appalachia has a rich history, unique culture, and many assets to address health needs [9,10]. For example, communities within Appalachia tend to have high levels of social capital and strong social networks [9,11,12].

There is a dearth of health research focused on Appalachia [7,13], and thus, the Appalachian Translational Research Network (ATRN; https://appalachianresearchnetwork.org/) was established to promote the development and implementation of sound approaches to community engagement within research and to speed the translation of scientific discoveries into improved health throughout Appalachia. ATRN is comprised of nine core academic partners in Appalachia: the Integrated Translational Health Research Institute of Virginia, Marshall University, Penn State University, Ohio University, The Ohio State University, the University of Cincinnati, the University of Kentucky, Wake Forest School of Medicine, and West Virginia University. ATRN grew originally from centers with funding from the National Center for Advancing Translational Sciences (NCATS) through a Clinical Translational Sciences Award (CTSA) or the National Institute of General Medical Sciences through an Institutional Development Award (IDeA). As part of this funding, each institution has prioritized community partnerships, adhering to community engagement principles, within translation [14].

Among its activities, ATRN hosts an annual Health Summit. The 10th Summit (held online September 22–23, 2020) was entitled “Community-Engaged Research in Translational Science: Innovations to Improve Health in Appalachia.” Originally planned to be in-person, the Summit was held as a videoconference due to the COVID-19 pandemic. The Summit was designed to:

1. Highlight current research to understand and reduce health disparities among diverse populations within Appalachia;
2. Identify innovative approaches to community-engaged research and translational science to improve the health of these populations;
3. Develop new and strengthen existing research partnerships, networks, and mentoring relationships to advance these approaches within the region;
4. Establish and disseminate priorities for future Appalachia-focused research.

We describe the Summit planning process and implementation, and highlight some of the research presented. Crosscutting themes emerged from notes taken by Summit planners and session moderators and review of session slides. Themes were confirmed by the Summit planning committee through iterative group review.

## Summit Planning and Coordination

The ATRN Health Summit was hosted by the Wake Forest Clinical and Translational Science Institute and accredited for health professionals by Wake Forest’s Office of Continuing Medical Education and Northwest Area Health Education Center (NWAHEC). A Summit planning workgroup comprised of representatives of the core academic institutions, and four community organization partners (12 members) oversaw the planning of the Summit. They developed and disseminated the call for abstracts for presentations, developed the program, selected and invited plenary speakers, and reviewed submitted abstracts. Concurrent sessions were organized thematically by accepted abstracts.

The registration fee was low to keep the Summit affordable for researchers, students, and community members. Community organizations presently or previously partnering with academic institutions were invited to participate in the Summit.

Of the 141 participants, 94 came from universities (across 20 different institutions, including one historically black college/university), 41 from community organizations (18 unique institutions), and six from the National Institutes of Health. Most of the university participants (67 out of 94, or 71%) came from the nine core partner organizations. The vast majority of participating organizations, whether from universities or community organizations, came from within the Appalachian Region: 17 of the 20 academic institutions and 16 of the 18 community organizations. Community organizations were reached through various networks and listservs, including established networks with the nine core ATRN academic partners.

The NWAHEC provided the Summit’s virtual platform. Pre-conference testing helped determine preferred videoconferencing platforms and timing, which included two half days of programming (Day 1: 9:45 am–12:35 pm; Day 2: 9:45 am–1:00 pm).

## Summit Program

Summit objectives were shared in a “Call for Abstracts” through the ATRN network. The planning workgroup reviewed abstracts, placed accepted abstracts into concurrent sessions, and titled the sessions based on the context of the abstracts. A total of 39 abstracts were selected for oral presentation and organized into 12 concurrent sessions. Sessions intentionally highlighted the work of early-career investigators. Plenary speakers were chosen based on previous Summit topic suggestions and represented community-academic partnerships conducting research pertinent to ATRN mission. Summit plenaries and concurrent sessions and presentations are outlined in Table 1. Research highlights that were representative of ATRN-focused areas are described below.

**Table 1.** “Community-engaged research in translational science: innovations to improve health in Appalachia”: plenaries, concurrent sessions, presentations, and authors

Plenary or concurrent session	Title	Authors
Plenary	*Introduction to the Integrated Translational Research Network of Virginia (iTHRIV)*	Hosig K., Virginia Tech
	*Exploring Influences of COVID-19 Messaging on Behavior in Virginia*	Cook N, Virginia Tech Friesen MA, INOVA Health Silverman R, Virginia Tech Pomfrey Wells E, University of Virginia at Wise Wenzel S, Virginia Tech
Innovative Approaches to Diabetes Prevention and Control	1. *Building an Effective Nutritional Handout for Underserved Appalachian Ohio*	Gaston S, Ohio University Heritage College of Osteopathic Medicine
	2. *Barriers Encountered Transitioning to Viral Testing with Rural Participants During a Pandemic*	Lewis C, Faulds E, Ohio State University
	3. *Using Digital Storytelling to Teach Healthcare Providers and Educators About Appalachian Culture*	Beverly E, Ohio University Heritage College of Osteopathic Medicine Love M, Ohio University
Social Determinants of Health: Families, Youth, and Race	1. *A Local Community-Partnered Project to Understand the Social and Health Needs of Women at the Transition to Adulthood*	Ballard P, Wake Forest School of Medicine Cannady J, Grigg M, Hill A, Davenport D, Woodruff R, Forsyth Futures
	2. *COVID-19 Impact on Mental Health, Lifestyle, and Substance Use Behaviors in a Cohort of Adolescent Boys in Ohio*	Tetreault E, Kenyon College
	3. *Facilitators and Barriers to Providing Breastfeeding and lactation Support to Families in Appalachia: Perspectives on Lactation Professional and Supporters*	Seiger E, Martin S, University of North Carolina Chapel Hill
	4. *Reaching the Hispanic Community about COVID-19 through Existing Chronic Disease Prevention Programs*	Calo W, Murray A, Penn State University
Innovative Methods to Translational Research: Part 1	1. *Life Through Their Lens: An Adaption of Photovoice for Community-Engaged Research in Amish and Mennonite Communities*	Thomas M, Ohio University Heritage College of Osteopathic Medicine
	2. *Detecting Radon Using Low-Cost detectors, Citizen Science, and Geology: The RADAR Project*	Stanifer S, Conley N, Rademacher K, Hoover A, Wolfe A, Hahn EJ, University of Kentucky Gross DA, St. Claire HealthCare, Northeast Kentucky AHEC Owens K, Rockcastle Regional Medical Center
	3. *BerryCare: A Sustainable Community-Academic Nutrition-Based Collaborative program to Promote Well-Being in Older Adults*	Brewer D, Koempel A, Stephenson T, Plasencia J, University of Kentucky
Food Insecurity Across the Lifespan	1. Persistence of Healthy Eating in Women After Participation in WIC: A Work-in-Progress Study	Martinez A, Ohio University Heritage College of Osteopathic Medicine
	2. BerryCare: Food Insecurity and the Role of Memory	Koempel A, Plasencia J, Stephenson T, Brewer D, University of Kentucky
	3. Site-Level Perceptions of Factors that Facilitate and Hinder the Success of Healthy Food Access Programs in Appalachian Ohio	Krzyzanowski Guerra K, Hanks D, Plakias Z, Castillo V, Carson C, Huser S, Redfern T, Barbaree J, Ali S, Garner JA, Ohio State University
Substance Use: Collaboration, Partnership, and Prescribing	1. Public Access to Withdrawal Management Services in a US Epicenter: A Single-Site Retrospective Study of Clinical Health Outcomes	Vissman A, Talbert House
	2. A Retrospective Analysis of Health Determinants Associated with Opioid Prescribing Rates	Pelini C, Ohio University Heritage College of Osteopathic Medicine
	3. Harnessing the Power of Peer Navigation and mHealth to Reduce Health Disparities in Appalachia	Rhodes SD, Mann-Jackson L, Alonzo J, Garcia M, Wilkin AM, Reboussin BA, Wake Forest School of Medicine Tanner AE, University of North Carolina Greensboro Del Toro A, Weil P, Western North Carolina AIDS Project
Creative Nutrition Interventions: Providers, Texting, and Families	1. Qualitative Description of Geographic and Family Dietary Norm Influence on Patient-Caregiver Dyad Dietary Patterns	Koonmen LA, Chung ML, Key KV, Mudd-Martin G, University of Kentucky
	2. Affective Place-Based Text Messaging May Be an Effective Approach to Improving Health Outcomes in Rural Appalachian Counties	Gillespie R, University of Kentucky
	3. Rural Obesity Medical Education for Primary Care in West Virginia: A Needs Assessment	Hernandez-Pachon M, Davisson L, Haggerty T, West Virginia University
Plenary	Food Insecurity and Health: From Clinical Screening to Community Engagement	Best S, HOPE of Winston-Salem Montez K, Palakshappa D, Zimmer R. Wake Forest School of Medicine
Successes in Patient Navigation and Telemedicine to Improve Health and Well-Being of Populations Living in Appalachia	1. Rural Patient Navigation: Addressing Barriers to Quality Cancer Care	Copus E, Wake Forest Baptist Health
	2. Patient Navigation and Cancer-Related Care: Policy Solutions to Improve Access to Pennsylvania’s Complex System of Care	Lengerich E, Penn State University
	3. Impact of Telemedicine in Acute Stroke Patients with Large Vessel Occlusions	Rawson J, Adcock A, West Virginia University
The Diabetes Epidemic: An Examination of What is Happening in Appalachia	1. Barriers Encountered Transitioning to Virtual Testing with Rural Participants During a Pandemic	Lewis C, Faulds E, Ohio State University
	2. Barriers and Facilitators in Caring for Women with Gestational Diabetes in Rural Appalachia	Chertok I, Silk J, Kulasa K, Ohio University
	3. The Impact of COVID-19 on Food Insecurity among Food Insecure Diabetic Patients in a Family Medicine Residency Practice	Wang H, Ohio Health Riverside Family Practice
	4. Complete Health Improvement Program: Associations of Whole Foods, Plant-Based Nutritional Intake and Short- and Long-Term Outcomes	Rowane M, Chavan B, Drozek D, Ohio University Heritage College of Osteopathic Medicine
Social Determinants of Health: The Role of Geography	1. Adverse Social Determinants of Health among Emergency General Surgery Patients in Rural Appalachia and Urban/Suburban Ohio: Results from a Feasibility Study	Baselice H, Ohio State Wexner Medical Center
	2. Using an Implementation Research Framework to Identify Facilitators and Barriers to Physical Activity and Weight Loss in Appalachia	Turner T, West Virginia University
	3. Gender Bias in the Assessment of New Onset Non-Traumatic Chest Pain: A Rural vs Urban ED Comparison	Tolbert A, Nemade D, Murphy C, Raines JA, Wehner P, Shuler FD, Marshall University
Food Insecurity: Across Communities	1. What a City Eats: Examining Dietary Preferences of Families Living in Communities at High Risk for Food Insecurity	Montez K, Wake Forest School of Medicine Best S, HOPE of Winston-Salem
	2. Relationships between Healthy Food Access Program Use, Diet, Health, and Food Security Status in Appalachian, Ohio during COVID-19	Garner J, Plakias Z, Castillo V, Hanks D, Carson C, Huser S, Redfern T, Barbaree J, Ohio State University
	3. Farm To You: An Innovative and Community-Engaged Strategy for Health Systems to Address Food Insecurity in Patients with Chronic Disease	Morton-Eggleston E, Moerschel S, Toolan C, Fiore N, West Virginia University
	4. An Exploration of Nutrition Needs and Barriers of Uninsured Clients of Free Clinics in Western North Carolina	Jameson E, Nunnery D, Appalachian State University
Innovative Methods to Translational Research: Part 2	1. Developing COVID-19 Intervention Strategies in Rural Appalachia: Using Biomedical Informatics to Identify, Understand, and Respond to Changing COVID-19 Patterns	Gurcan M, Driscoll DL, Wells E, Talbert JC, Wake Forest School of Medicine
	2. Virtual Focus Groups as an Effective Tool for Community Data Collection During a Pandemic	Cook N, Wenzel S, Jiles K, Markwalker T, Virginia Tech
	3. Addressing Inequalities in Occupational Therapy Through 3D Printing	Powell J, Wake Forest School of Medicine
Advances in Health Care and Disease Management: Lung Disease, Neurology and Asthma	1. Neurological Complications of COVID-19: A Systematic Review of Literature	Adcock A, West Virginia University
	2. Palliative Care Intervention for Rare Advanced Lung Diseases in Underserved Appalachia: A Pilot Randomized Controlled Trial	Piamjariyakul U, Smothers A, Petitte T, Young S, Morrissey E, West Virginia University
	3. The BREATH Study: Using Breath biomaRkers to understand Environmental contributions to Asthma in THe Appalachian Region of Kentucky	Sturgill J, University of Kentucky
	4. The Role of the Gut Microbiome in Inflammation and Pain in Orthopaedic Conditions	Dahshan D, Workman A, Perdue J, Gallagher N, Schmicker T, Shuler FD, Marshall University School of Medicine
Plenary	Opioid Research Consortium of Central Appalachia (the ORCCA)	Hagaman A, Pack R, East Tennessee State University Work S, Horn K, Virginia Tech

### Exploring Influences on COVID-19 Messaging on Behavior in Virginia

A mixed-methods study conducted by Cook and colleagues sought to understand how health behaviors might influence COVID-19 infection rates in Virginia early during the pandemic. The study was advertised on social media and online, and data were collected online. Thirty-one percent of participants (n = 1041) lived in an Appalachian county. A high proportion reported changing their behaviors to be safer; for example, 95% said they practiced social distancing and 89% said they washed their hands more often. However, 121 participants (12% of the sample) reported that they believed one or more false or inaccurate messages, most commonly that COVID-19 was developed as a bioweapon. These participants were more likely to self-identify as Republican, be 18–24 years old, and have a high school diploma/GED or below. The investigators subsequently convened several virtual focus groups with community subgroups including African Americans and young adults. Themes that emerged included confusing or contradictory advice about COVID-19 risk and transmission and “pandemic fatigue.”

### Social Determinants of Health: Families, Youth, and Race

Ballard and colleagues described preliminary findings from a pilot study examining social and health needs and assets among primarily African American women transitioning to adulthood (ages 18–29). The team conducted 15 semi-structured in-person interviews of African American women residents of Northeast Winston-Salem, a structurally disadvantaged urban area within Appalachia. Themes that emerged included the following: wellness defined holistically to encompass both mental and physical health; mental health identified as a more salient topic than physical health; of their most prominent concerns and structural barriers to health and wellness that included inadequate medical insurance and reduced access to care. In this study, the transition to adulthood emerged as an under-recognized developmental period for health and wellness, and resources were identified as less available compared to people in other life stages.

### Substance Use: Collaboration, Partnership, and Prescribing

Vissman and colleagues examined referral and discharge patterns at the first public access, clinically managed withdrawal management treatment center in the Cincinnati area. Cincinnati is a national epicenter for opioid overdoses. Noting a dearth of reports on patterns of referral to and from clinically managed withdrawal management services supported by the American Society of Addiction Medicine, this team performed a retrospective chart review from May 2018–2019 of 588 unique admissions to one withdrawal management treatment center in Cincinnati. Most clients (73%) were on Medicaid, and the majority (68%) were men. Of the 109 individuals admitted, nearly all (98%) were admitted for residential services. About 40% had successful discharge referral status (confirmed housing and plans for medically assisted treatment at discharge).

Gay, bisexual, other men having sex with men (GBMSM) and transgender persons are at increased risk for HIV, sexually transmitted infections (STIs), and hepatitis C. Although some services exist, including syringe services, health disparities remain, as does a need for culturally congruent interventions for racially/ethnically diverse GBMSM and transgender persons in rural Appalachia. Rhodes and colleagues described the early phases of a novel community-based participatory research study to integrate mHealth outreach and peer navigation strategies to increase rates of testing for HIV, STIs, and hepatitis C and engagement in care; and use of related prevention and care services, including syringe services. In this ongoing NIH-funded project, community health leaders and their social network members are being randomized to mHealth social media/peer navigation intervention arm or a delayed intervention arm.

### Food Insecurity and Health: From Clinical Screening to Community Engagement

Best and colleagues described two pilot clinic-based interventions aimed to reduce food insecurity. The first program, “Food is Health,” screens all patients in an urban outpatient clinic for food insecurity with a validated 2-item screening tool [15]. Responses are then incorporated into the provider workflow and captured and flagged in the electronic health record (EHR) if responses indicate any possible food insecurity. For those with food insecurity, a navigator provides food for three meals for a family of four through an on-site food pantry and facilitates referrals to other food resources. Preliminary data suggest that over two years, the program has significantly reduced food insecurity among clinic patients, with caregivers having a significantly lower odds of reporting food insecurity after the program was begun (OR = 0.56, 95%CI 0.48, 0.66; p < 0.001).

### Successes in Patient Navigation and Telemedicine to Improve Health and Well-Being in Populations Living in Appalachia

Cancer patients in Appalachia are often isolated and face transportation challenges that can significantly affect prevention, diagnosis, care, and recovery. Copus and colleagues presented population health navigation as a pilot strategy to address these challenges. Three navigators were hired, trained, and supported to focus on the needs of rural African American and Latinx patients. Using EHR data of rural patients, the team identified treatment logistics and financial/insurance matters to be the primary barriers to care. Thus, nearly 65% received support for transportation and 47% for parking. Overall, 59% of patients were referred to supportive care services, including mental health counseling, financial resources, or housing support. Based on study findings, financial barriers will be assessed in future research.

Lengerich described engagement at the policy level to improve cancer care coordination in Pennsylvania. The Penn State Cancer Institute’s 28-county catchment area includes 19 Appalachian counties. Pennsylvania has a governor-appointed Cancer Advisory Board and a statewide Cancer Coalition (a workgroup with over 100 members).[16] Key recommendations from the workgroup to improve care coordination in complex settings included incorporating patient navigators into care pathways and classifying their services as reimbursable by insurance companies. The Coalition also called for standard criteria and training for patient navigators and more recognition of patient navigators’ value by employers.

### Food Insecurity: Across Communities

Jameson and colleagues presented data regarding barriers to nutrition and the need for nutrition education in a region of Appalachia in western North Carolina. The team surveyed participants (n = 202) in waiting rooms at two free county health clinics. Participants indicated an interest in receiving health education (overall health, weight loss, diabetes, blood pressure, and heart disease) and in using mHealth, social media, and other online resources (e.g., videos) as delivery channels. Most participants expressed interest in access to local produce (86%) and recipes (71%). While many participants noted assets, such as access to Wi-Fi and food pantries, they also noted barriers, such as irregular computer access and needing guidance on healthy meals preparation. Lack of housing or relevant resources (e.g., appliances, money, or food) were cited by 23% and 38% of clients at each clinic, respectively.

### Innovative Methods to Translational Research

Radon, a naturally occurring radioactive gas, is the second-leading cause of lung cancer, and the risk of lung cancer is higher among those exposed to both radon and tobacco smoke. Despite the known risk, few people assess their homes for radon. Stanifer and colleagues described efforts to increase radon detection in Kentucky harnessing “citizen scientists.” Trained community members measured radon daily in their homes, using low-cost digital radon detectors, and texting results to investigators. Because of the pandemic, participants were recruited via social media, gave informed consent virtually using REDCap, and were trained remotely via videoconferencing. Among four counties with higher radon potential, the team has tested about 10% of homes. Affordable radon mitigation is the next step in this ongoing project.

CTSA hubs across Appalachian are collaborating to share COVID-19-related data to develop models better reflecting the region’s characteristics. For example, Gurcan and colleagues are determining social and emotional predictors of social distancing behaviors; identifying possible periods of higher parental stress using family demographics and related child welfare indicators; and assessing sleep habits and mental health factors before, during, and after “waves” of social distancing. These data will be used to design interventions to promote greater adherence to social distancing, improve coping skills (e.g., receiving SARS-CoV-2 vaccination), and reduce individual and family stress for future waves of COVID-19 infections. In addition, these data could help to reduce COVID-19 spread and mortality in Appalachia; improve access to limited resources (e.g., personal protective equipment and telemedicine); and enhance effectiveness of communications regarding future outbreaks.

## Emergent Summit Themes to Guide Research

Nine themes emerged from the 39 presentations that provide guidance for future Appalachian-focused research to address the myriad of needs in the region (Table 2).

**Table 2.** ATRN annual health summit 2020 crosscutting research needs and themes to guide future Appalachian-focused research

1. Social determinants of health must be better understood and addressed
2. Community-academic partnerships that are mutually beneficial and build capacity are needed
3. Hidden communities and populations must be prioritized, including the following: racial/ethnic, sexual, and gender minorities; immigrants; adolescents and young adults; and non-English speakers
4. A profound need exists for both knowledge generation and the development of context-specific strategies that consider the heterogeneity of the Region, including rural and urban settings
5. Further development of innovative methods aligned with translation and community engagement is needed
6. Community strengths and assets can and should be harnessed to improve health and well-being
7. mHealth and telemedicine are promising approaches to reach diverse rural communities
8. Bioinformatics has potential to improve health and well-being in Appalachia
9. As new needs and priorities emerge, existing needs and priorities cannot be ignored

First, addressing individual risk factors associated with morbidity and mortality in Appalachia is critical, but efforts must include understanding and intervening on upstream social determinants of health (e.g., education, employment, transportation, housing, and access to health care). Second, mutually beneficial community-academic partnerships that build capacity are needed. Strengthening these partnerships will enable them to conduct rigorous and engaged research to improve the health and well-being of communities and populations within Appalachia. Third, there is a need to acknowledge Appalachia’s heterogeneity and address the needs and priorities of “hidden” and thus neglected communities. For example, Latinx persons are a growing proportion of the Appalachian population, and profound disparities exist within Appalachia by racial/ethnic, sexual, and gender minority status. Fourth, Appalachia is large and diverse, with rural and urban areas that have different needs, priorities, and assets, and are in different states. These differing state and municipal contexts can greatly influence health and well-being. For example, six states within Appalachia have yet to expand Medicaid. Fifth, although the rationale for translation and community engagement is well documented and methods such as photovoice [17], empowerment-based community forums [18], and citizen science [19] are well developed, we still lack methodologic innovations aligned with translational science and community engagement. Sixth, community strengths and assets (e.g., existing social networks and cohesion) should be harnessed to improve community health and well-being in Appalachia. For too long, a deficits approach has been applied when considering health and well-being within the region; there has been insufficient focus on harnessing assets, such as existing social networks and cohesion. Seventh, mHealth and telemedicine are promising approaches to reach diverse communities, where smartphones now allow members of under-resourced communities to access free Wi-Fi in public spaces. Eighth, bioinformatics has potential to use clinical data and key stakeholder feedback to further translation of research into practice and inform prevention and intervention strategies that improve health and well-being in Appalachia. Finally, as new needs and priorities emerge (e.g., the COVID-19 pandemic), research questions and methods must evolve to meet them.

## Summary

The 2020 ATRN Health Summit brought together community, academic, and NIH stakeholders to highlight innovative community-engaged research focused on multiple health disparities in Appalachia, including chronic diseases, social determinants of health, environmental health, substance use and abuse, and infectious diseases among diverse communities and populations. Novel approaches, strategies, and methods aligned with translational science and community-engaged research were highlighted and critical areas of focus for future work were outlined. The themes that arose from the Summit highlight research need guidance for future Appalachian-focused research.

## Future Directions

Emergent themes will be used to guide future ATRN Health Summits and the research activities of various community-academic partnerships across the region. Collaborations established across the core institutions and community partners will explore identified needs, such as hidden populations and utilization of bioinformatics and community engagement. Funding needs and priorities can be articulated to organizations with a focus on Appalachia and health disparities, with a focus on the strengths of the Appalachian Region.
